# Efficacy and safety evaluation of black ginseng (*Panax ginseng* C.A. Mey.) extract (CJ EnerG): broad spectrum cytotoxic activity in human cancer cell lines and 28-day repeated oral toxicity study in Sprague-Dawley rats

**DOI:** 10.1186/s12906-022-03522-3

**Published:** 2022-02-16

**Authors:** Jin-Sung Park, Seung-Hyun Kim, Kang-Min Han, Yun-Soon Kim, Euna Kwon, Se-Hee Paek, Yong-Ki Seo, Jun-Won Yun, Byeong-Cheol Kang

**Affiliations:** 1 Department of Experimental Animal Research, Biomedical Research Institute, Seoul National University Hospital, Seoul, Republic of Korea; 2 Graduate School of Translational Medicine, Seoul National University College of Medicine, Seoul, Republic of Korea; 3 Department of Pathology, Dongguk University Ilsan Hospital, Goyang, South Korea; 4 Food R&D Institute, CJ CheilJedang Corp., Suwon, Republic of Korea; 5 Department of Biotechnology, The Catholic University of Korea, Bucheon, Republic of Korea; 6 Biomedical Center for Animal Resource and Development, Seoul National University College of Medicine, Seoul, Republic of Korea; 7 Designed Animal and Transplantation Research Institute, Institute of GreenBio Science Technology, Seoul National University, Pyeongchang-gun, Gangwon-do Republic of Korea

**Keywords:** Black ginseng, Cytotoxic activity, Ginsenoside Rg5, Preclinical safety evaluation, Repeated oral toxicity

## Abstract

**Background:**

Ginseng (*Panax ginseng* C.A. Mey.) has been used as a valuable ingredient in traditional medicine for thousands of years mostly in Asian countries due to its therapeutic effects in various diseases. Among the processed ginseng products, black ginseng is produced by a repeated steaming and drying process of ginseng roots and has been known for its superior efficacy based on high accumulation of minor ginsenosides as recently discovered. Despite its popularity and increasing use, the toxicity information on black ginseng still remained largely lacking, raising safety concerns. This study was therefore carried out to determine the repeated oral toxicity of black ginseng extract (BGE; CJ EnerG) with evaluation of cytotoxic activity as validation of its pharmacological activity for toxicity testing.

**Methods:**

Prior to the toxicity test, we examined the cytotoxicity of BGE in six cancer cell lines derived from distinct human tissues in comparison with red ginseng extract (RGE), ginsenosides Rg5 and 20(S)-Rg3, and then assessed 28-day repeated oral toxicity in Sprague-Dawley (SD) rats using daily administration of up to 2000 mg/kg BGE.

**Results:**

BGE showed higher cytotoxicity than RGE in all the cell lines used in this study. Interestingly, the efficacy of BGE closely resembled the cytotoxic pattern of Rg5, suggesting Rg5 as the main effector in the cytotoxic activity of BGE. During the toxicity study, BGE-treated groups showed no noticeable abnormality in clinical signs, body weight gain, food and water consumption and urinalysis. Furthermore, hematological, serum biochemical and histopathological analyses did not find any BGE-related toxicity.

**Conclusion:**

Our findings demonstrated that BGE has broad-spectrum in vitro cytotoxic activity, and that NOAEL of BGE in SD rats is > 2000 mg/kg, providing the essential safety information for human consumption.

**Supplementary Information:**

The online version contains supplementary material available at 10.1186/s12906-022-03522-3.

## Background

*Panax ginseng* is a perennial plant belonging to genus *Panax* under the *Araliaceae* family, which consists of about 14 species including *P. ginseng* C.A. Mey. (Korean ginseng), *P. quinquefolius* L. (American ginseng), and *P. notoginseng* (Burkill) F.H. Chen (Chinese ginseng) [1]. Ginseng roots have been one of the most important and valuable ingredients mainly in Asian folk medicine due to their pharmacological benefits. Consistently, a line of recent studies revealed therapeutic activities against a variety of clinical symptoms and diseases such as tumorigenesis, memory loss and cognitive dysfunction, neurodegenerative diseases, diabetes, cardiovascular diseases, abnormal blood pressure, and male sexual dysfunction [1, 2].

Ginseng contains biologically active components known as ginsenosides [3]. Many ginsenosides are exclusively found in ginseng and reported to account for a large part of ginseng-mediated medicinal effects [4, 5]. Until now, approximately 200 major and minor ginsenosides have been associated with ginseng [6], of which 38 have been isolated from Korean ginseng [2].

Along with simple use of fresh ginseng roots, several processing methods have been traditionally applied to increase preservability and effectiveness; White ginseng is the dried product of peeled roots, while red ginseng is produced through steaming and drying of fresh roots without peeling skin. Preparation of black ginseng requires further effort and time of repeating the steaming and drying cycle. Interestingly, these ginseng products have been observed to be significantly different in the range of applicable diseases and efficacy according to the processing methods [7], and recent studies discovered that the kinds and concentration of ginsenosides may be responsible; white ginseng is rich in main ginsenosides including Rg1, Rb1 and Rb2, while minor ginsenosides such as Rg3, Rg5 and Rk1 become more abundant in red ginseng and, to even higher levels, in black ginseng due to accumulation during processing [8, 9].

Black ginseng has been known for its superior pharmacological activity, to which accumulation of minor ginsenosides plays as the main contributing factor [10]. The repetitive processing facilitates structural transformation of Rb1, leading to accumulation of Rg3 and Rg5 [11]. Both Rg3 and Rg5 have been shown to possess high biological activities [12], among which anti-cancer activity has been well documented in several cancer cell lines and xenograft mouse models [13–18].

Ginseng has been generally acknowledged as safe based on its clinical use in traditional medicine and the results of toxicity studies [19–21]. Among the processed ginseng products, safety of red ginseng has been well investigated, establishing essential toxicity parameters including LD50 and NOAEL in model systems [22–26]. On the contrary, safety information on black ginseng has been limited despite its growing popularity in commercial markets, raising safety concerns; only one study reported acute oral toxicity for single oral administration of black ginseng [27], but to date the toxicity profile associated with its repetitive use has not been available. In this study, we therefore evaluated the subacute oral toxicity of black ginseng extract (BGE; commercially known as CJ EnerG) in SD rats. Prior to the toxicity study, we verified the pharmacological validity of BGE by examining cytotoxic activity in six human cancer cell lines in comparison with red ginseng extract (RGE). Our results demonstrated that compared to RGE, BGE had higher cytotoxic activity in all cancer cell lines tested in this study largely due to its high contents of Rg5, and did not cause recognizable test substance-associated toxicity in vivo.

## Methods

### Test substance

Four-year-old *P. ginseng* C.A. Mey. roots cultivated in the Geumsan area (Chungcheongnam-do, South Korea) were purchased from Geumsan Susam center (Geumsan, Chungcheongnam-do, South Korea) and used as the raw material for production of the test substances. Black ginseng (BGE; CJ EnerG) and red ginseng extracts (RGE) were prepared, analyzed for the levels of ginsenoside Rb1, Rg1, Rg3 and Rg5, and then provided for the study by CJ CheilJedang Corporation (Suwon, Gyeonggi-do, South Korea) as previously described [28]. Ginsenoside 20(S)-Rg3 (≥ 98.0%) was purchased from Ambo Institute (Daejeon, Korea) and Rg5 (≥ 98.0%) from Chengdu Biopurity Phytochemicals Ltd. (Chengdu, Sichuan, China). Paclitaxel (PTX) and all other chemicals used in this study were obtained from Sigma Aldrich (St. Louis, MO, USA) unless otherwise stated. Voucher specimens (SNUH 14016–8 and 14,016–9) have been deposited at Department of Experimental Animal Research, Biomedical Research Institute, Seoul National University Hospital. The selection of raw materials and preparation of test substances was performed in compliance with the relevant national guidelines of South Korea including Health functional food Act (Act No. 6727, South Korea) and Health functional food code (No. 2018–12, Ministry of food and drug safety, South Korea).

### Cell culture

Cytotoxic activity of BGE was assayed using 6 cell lines derived from human carcinomas of different origin; A-431 (skin; CRL-1555, ATCC, Manassas, VA, USA), A549 (lung; CCL-185, ATCC), HT-29 (colon; HTB-38, ATCC), NCI-N87 (stomach; CRL-5822, ATCC), Capan-1 (pancreas; HTB-49, ATCC) and HepG2 (liver; 88,065, Korean Cell Line Bank, Seoul, South Korea). In order to solely compare the effect of treatment compounds without introducing any compounding factors by use of different media, all cell lines were grown in RPMI 1640 media (Gibco, Life technologies Corporation, Grand Island, NY, USA) supplemented with 10% FBS (16000–044, Gibo), 23.8 mM NaHCO_3_, 100 units/mL penicillin, 100 μg/mL streptomycin and 250 ng/mL amphotericin at 37 °C with 5% CO_2_ and 100% relative humidity in a CO_2_ cell culture incubator (Heracell 150, Waltham, MA, USA).

### Cytotoxicity assay

Cells were plated at 1 × 10^4^/well in 96 well plates in triplicate and grown for 24 h. PTX was dissolved in DMSO, while BGE, RGE, 20(S)-Rg3 and Rg5 in ddH_2_O. Cells were treated with either PTX at 1:100 or one of the aforementioned compounds at 1:10 in the culture media for 24 h in the CO_2_ incubator. The respective vehicles were used for negative control. Final concentrations used for treatment were 312.5, 625, 1250, 2500 and 5000 μg/mL for BGE and RGE, 31.25, 62.5, 125, 250 and 500 μg/mL for Rg5 and 20(S)-Rg3, and 0.64, 3.2, 16, 80 and 400 nM for PTX. After replacement with fresh media the next day, the cells were overlaid with 50 μL of 2 mg/mL MTT and incubated for 4 h in the CO_2_ incubator. Following a brief centrifuge, media was removed and formazan crystal formed in each well was dissolved with 100 μL DMSO. Optical density (OD) was determined at 540 nm using a Multiskan GO spectrophotometer (Thermo Fisher Scientific, Waltham, MA, USA) and percentage of cell viability was calculated by comparing average OD of the treated wells and their respective negative controls.

### Animals

Six-week-old male and female SD rats were purchased from Orient Bio Inc. (Sungnam, Gyeonggi-do, South Korea) and acclimatized for a week before study initiation. All animals were housed in an environment-controlled room at the AAALAC International-accredited animal facility (#001160) in Biomedical Research Institute of Seoul National University Hospital with free access to sterilized laboratory rodent diet (2918C, Harlan Laboratories Inc., Indianapolis, IN, USA) and autoclaved water. All experiments were approved by the Seoul National University Hospital Institutional Animal Care and Use Committee in accordance with Guide for the Care and Use of Laboratory Animals, 8th edition. The animal study was carried out in compliance with the Animal Research: Reporting In Vivo Experiments (ARRIVE) guidelines (https://arriveguidelines.org/).

### Subacute oral toxicity study

Twenty eight-day repeated dose oral toxicity study was conducted in accordance with OECD test guideline No. 407 [29]. Briefly, 7-week-old SPF SD rats (*n* = 10/gender/group) daily received oral administration of either 0, 500, 1000 or 2000 mg/kg BGE in 10 mL DW for 28 days. During the study period, all animals were monitored daily for clinical signs with body weight and food/water consumption measured once a week. In the last week of administration, ophthalmological examination was performed using indirect ophthalmoscopy and urine was analyzed using a urinalysis stick (Multistix 10 SG, Siemens, Munich, Germany) for pH, specific gravity, leukocytes, nitrite, protein, ketone body, urobilinogen, bilirubin, glucose and occult blood.

### Hematology and serum biochemistry

On completion of BGE administration, animals were deeply anesthetized using isoflurane and euthanized by exsanguination via the vena cava after blood sampling. Whole blood was collected in an EDTA tube (BD, Franklin Lakes, NJ, USA) and analyzed for white blood cell (WBC) count, red blood cell (RBC) count, hemoglobin (HGB) concentration, hematocrit (HCT), mean corpuscular volume (MCV), mean corpuscular hemoglobin (MCH), mean corpuscular hemoglobin concentration (MCHC), platelet (PLT) count, differential WBC count using an ADVIA2120i animal blood counter (Siemens Healthcare Diagnostics Ltd., Tarrytown, NY, USA). Prothrombin time (PT) and activated partial thromboplastin time (aPTT) were determined in plasma samples using an ACL-100 coagulation analyzer (Instrumentation Laboratory, Bedford, MA, USA).

Serum was separated from clotted whole blood by brief centrifugation and analyzed for blood urea nitrogen (BUN), total cholesterol (TC), total protein (TP), albumin, total bilirubin (TB), alkaline phosphatase (ALP), aspartate transaminase (AST), alanine transaminase (ALT), γ-glutamyl transferase (GGT), creatinine, triglyceride, glucose, albumin-globulin (A/G) ratio, K, Cl, Na, Ca and P using a Hitachi7180 automatic chemistry analyzer (Hitachi, Tokyo, Japan).

### Necropsy and histopathology

Necropsy was performed on all surviving animals after euthanasia. All major organs were examined for gross lesions, and heart, liver, lung, spleen, kidneys, adrenal glands, testes, ovaries, brain, pituitary gland and thymus were weighed before fixation in appropriate fixatives; testes and epididymides in Bouin’s solution (225 mg of picric acid dissolved in a mixture of glacial acetic acid, 37% formalin and DW at the ratio of 1:5:15.4), harderian glands and eyes in Davidson solution (a mixture of 95% ethanol, 10% formalin, glacial acetic acid and DW at the ratio of 3:2:1:3), and all other organs in 10% neutral formalin (GD Science, Gunpo, Gyeonggi-do, South Korea). All fixed organs from the negative control and 2000 mg/kg BGE groups were sectioned into 2–3 μm tissue slices after dehydration and paraffinization. Histopathological evaluation was performed on hematoxylin (Biognost, Zagreb, Croatia) and eosin (BBC Biochemical, Mt. Vernon, WA, USA)-stained tissue slices using bright-field microscopy (BX-51, Olympus Corporation, Tokyo, Japan).

### Statistical analysis

All data are represented as mean ± S.D. Statistical analysis was performed using one-way ANOVA followed by *post-hoc* Dunnett’s test in a SPSS software (version 22, IBM, Chicago, IL, USA). A *P* value less than 0.05 was considered as statistically significant.

## Results

### Black ginseng extract showed broad-spectrum cytotoxic activity with higher efficacy than red ginseng extract

Quantitative analysis of ginsenosides according to the published protocol [28] revealed that BGE used in this study was enriched with Rg3 and Rg5; the respective levels of Rb1, Rg1, Rg3 and Rg5 were 5.3 mg/g, 1.5 mg/g, 0.8 mg/g and 1.5 mg/g in RGE, and 1.5 mg/g, 0.1 mg/g, 5.2 mg/g and 20.6 mg/g in BGE, indicating that Rg3 and Rg5 in BGE were 6.5-fold and 14.1-fold higher than RGE, while RGE contained 14-fold and 3.5-fold more Rg1 and Rb1 than BGE. These results indicated the effectiveness of our BGE production process in conversion of ginsenosides, confirming high concentration of minor ginsenosides in BGE.

**Fig. 1:**
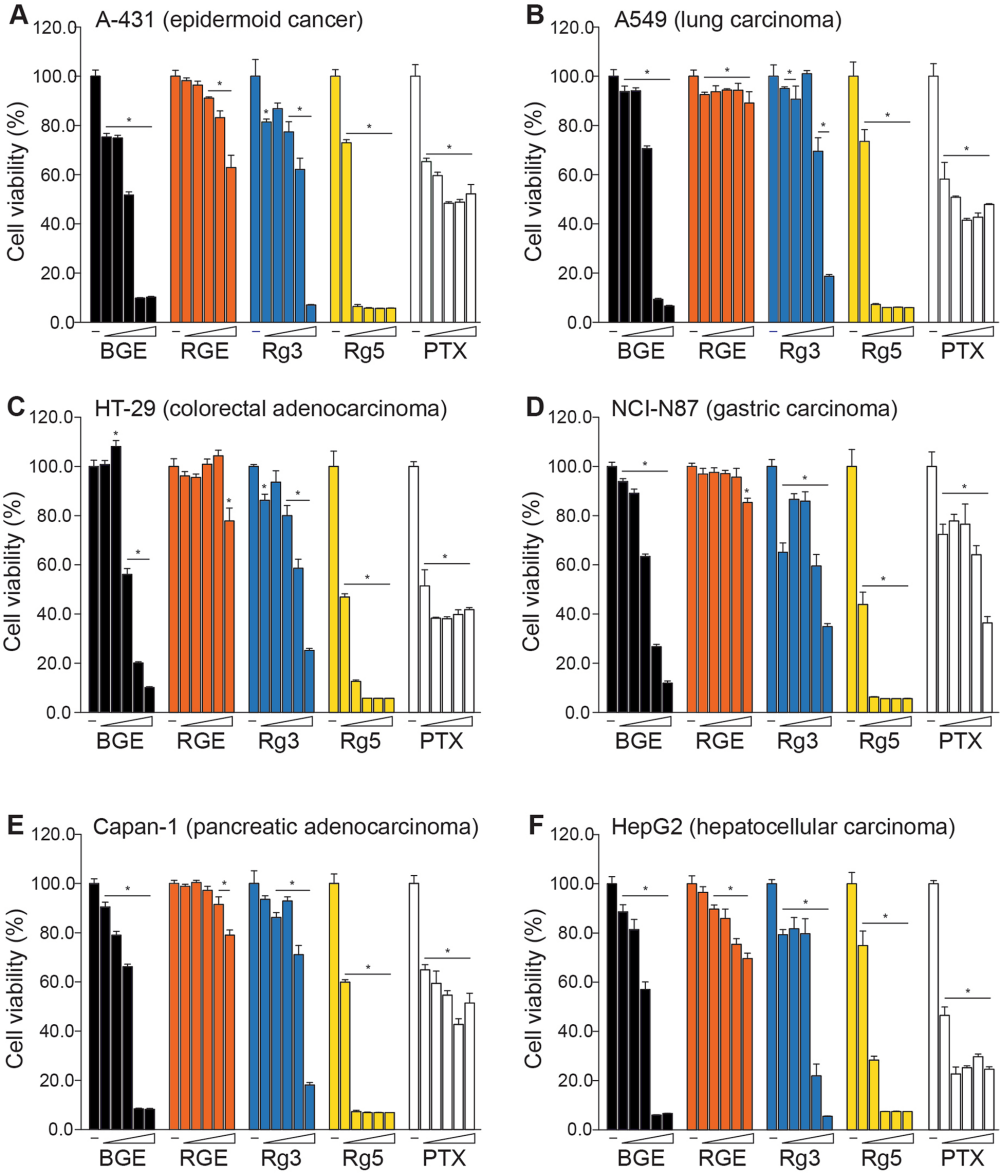
Cytotoxic activity of black ginseng extract (BGE) in human cancer cell lines. Cytotoxic effect of BGE was compared with red ginseng extract (RGE), ginsenoside 20(S)-Rg3 and Rg5 and Paclitaxel (PTX) in six human cancer cell lines with distinct tissue origins as indicated. Final concentrations used for treatment were 312.5, 625, 1250, 2500 and 5000 μg/mL for BGE and RGE, 31.25, 62.5, 125, 250 and 500 μg/mL for Rg5 and 20(S)-Rg3, and 0.64, 3.2, 16, 80 and 400 nM for PTX. *; *p* < 0.05 by one-way ANOVA followed by *post-hoc* Dunnett’s *t*-test

Black ginseng has been used in various diseases for its pharmacological effects [10], among which anti-cancer activity has been well documented in multiple studies [16, 30, 31]. Prior to testing its in vivo toxicity, we validated the efficacy of BGE prepared for this study by performing a series of cytotoxicity tests using six cancer cell lines derived from human carcinomas with different tissue origins; A431 (skin), A549 (lung), HT-29 (colon), NCI-N87 (stomach), Capan-1 (pancreas) and HepG2 (liver) cells. In the cytotoxicity assays, we employed purified 20(S)-Rg3 and Rg5 to dissect the contribution of Rg3 and Rg5 to the cytotoxic effect of BGE, and the chemotherapy agent paclitaxel (PTX) was used as a positive control (Fig. 1). When treated on the cancer cell lines, PTX exerted a marked cytotoxicity at low doses with its effect saturated above the doses over 16 ~ 80 nM. BGE significantly reduced viability of all cell lines from the lowest dose tested (312.5 μg/mL) in a dose-dependent manner with an exception for HT-29 cells which showed a visible decrease from 1250 μg/mL, resulting in complete cell death from 2500 μg/mL for A-431, A549, Capan-1 and HepG2, and 5000 μg/mL for HT-29 and NCI-N87. In comparison, RGE showed a milder degree of cytotoxic effect only at the high doses compared to BGE; RGE elicited cytotoxicity in HT-29, NCI-N87 and Capan-1 cells with significant cell death detected from 2500 μg/mL (Capan-1) or at 5000 μg/mL (HT-29 and NCI-N87), while A-431 (> 1250 μg/mL) and HepG2 (> 625 μg/mL) cells dose-dependently responded from the medium doses. Notably, RGE caused weak cell death in A549 cells regardless of the treated doses.

On comparing 20(S)-Rg3 with Rg5, Rg5 displayed higher cytotoxicity than 20(S)-Rg3 in all cell lines tested, suggesting a greater cytotoxic potency of Rg5. Interestingly, the cytotoxicity pattern of BGE closely resembled that of Rg5 at the doses containing equivalent concentration of Rg5 (5000 μg/mL and 2500 μg/mL of BGE contained 103.0 μg/mL and 51.5 μg/mL Rg5, respectively). The calculated IC50 was found between 1228.0 ~ 1461.4 μg/mL for BGE and over 5000 μg/mL for RGE, while Rg5 had approximately 6-fold lower IC50 than Rg3 for all the cell lines except for NCI-N87 (8.6 folds) and HepG2 (3.7 folds) (Table 1).

**Table 1:** IC50 for cytotoxic activity of black ginseng extract in human cancer cell lines

Test substance	IC50					
	A-431	A549	HT-29	NCI-N87	Capan-1	HepG2
BGE (μg/mL)	1302.1 ± 7.1	1461.4 ± 22.2^**^	1228.0 ± 19.9	1528.6 ± 55.2^**^	1453.4 ± 20.0^**^	1357.7 ± 30.3
RGE (μg/mL)	> 5000.0	> 5000.0	> 5000.0	> 5000.0	> 5000.0	> 5000.0
20(S)-Rg3 (μg/mL)	268.7 ± 7.4	262.9 ± 5.5	300.0 ± 5.0^*^	251.7 ± 1.0	269.2 ± 5.3	212.0 ± 20.3^**^
Rg5 (μg/mL)	39.2 ± 2.9	42.8 ± 8.5	50.0 ± 5.2	29.4 ± 0.6	41.6 ± 3.1	57.9 ± 2.6^**^
Paclitaxel (nM)	3.6 ± 0.3	4.0 ± 1.9	0.9 ± 0.3	118.8 ± 53.6^**^	21.5 ± 5.7	1.0 ± 0.1

*BGE* Black ginseng extract, *RGE* Red ginseng extract; *; *P* < 0.05 and * *P* < 0.01 compared to IC50 of A-431 by one-way ANOVA followed by *post-hoc* Tukey HSD multiple comparison test

### Black ginseng extract did not adversely affect the main physiological parameters in SD rats

We conducted a 28-day repeated oral toxicity study in SD rats using BGE with proven efficacy to assess its potential health risk. The combined amount of Rb1, Rg1, Rg3 and Rg5 in BGE used for toxicity study was determined to be 12.1 mg/g.

**Fig. 2:**
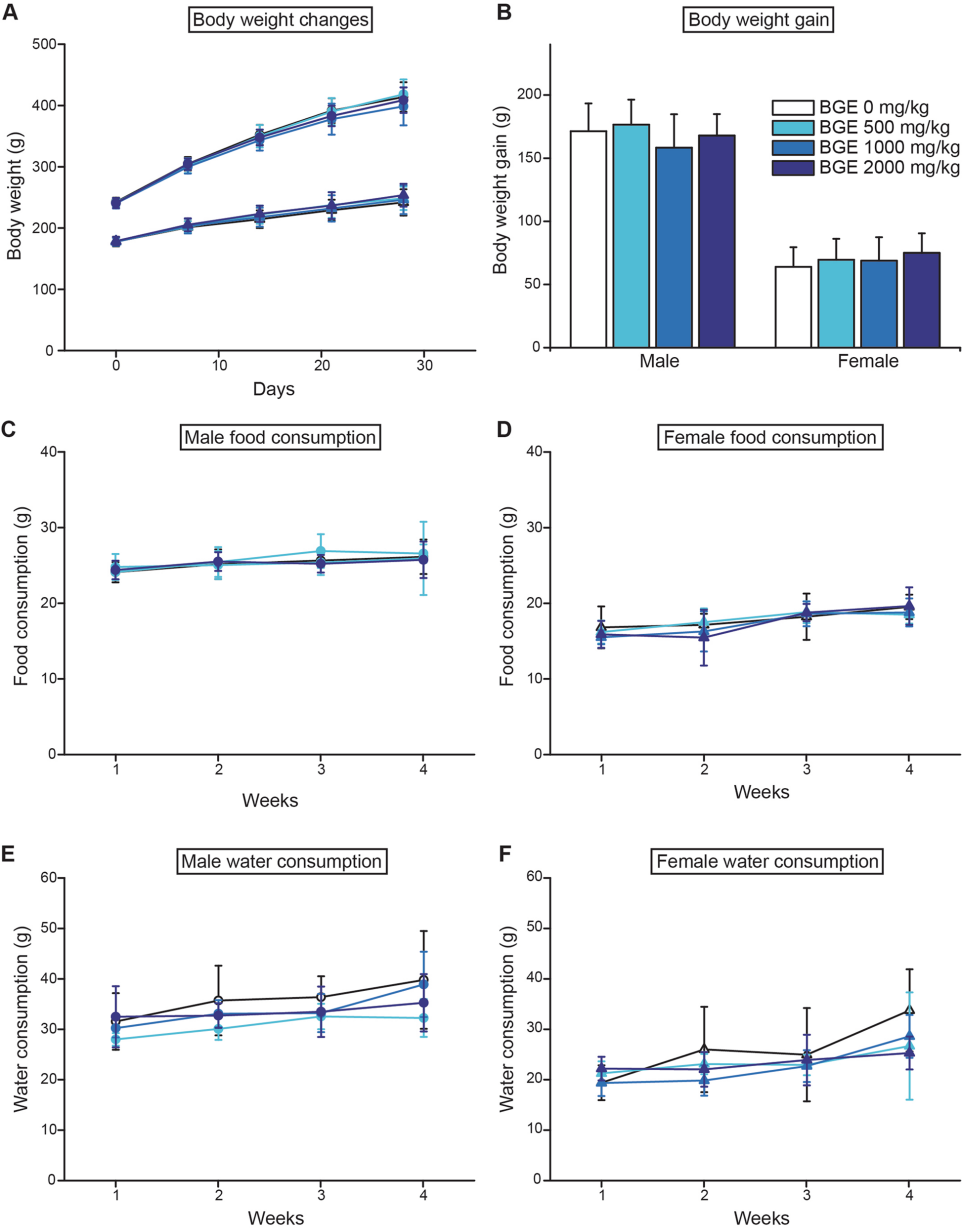
Changes in body weight, food and water consumption in Sprague-Dawley rats during 28-day oral administration of black ginseng extract (BGE). BGE-treated groups did not show noticeable difference from the control group in the indicated parameters. Circles; male and triangles; female. White, light-grey, dark-grey and black symbols for BGE-treated groups

During the whole study period, none of SD rats (*n* = 10/gender/group) showed any abnormal clinical signs or mortality (data not shown). Regardless of administered doses, no difference was observed among all groups in body weight gain and consumed amount of food and water (Fig. 2). The urinalysis (Supplementary Table 1) and ophthalmological examination (data not shown) conducted in the last week of the study did not find any test substance associated changes.

### Normal hematological and serum biochemical profiles in SD rats orally treated with black ginseng extract for 28 days

On completion of BGE administration for 28 days, whole blood was collected from the vena cava and analyzed for hematological parameters (Table 2). The percentage of monocytes was significantly increased in the male 2000 mg/kg group compared to the vehicle control, and remarkable reduction of aPTT was noted in all male groups treated with BGE. There was no notable change observed in all female groups.

**Table 2:** Hematological and serum biochemical parameters of SD rats orally treated with black ginseng extract for 28 days

	Dose of black ginseng extract (mg/kg) (male; *n* = 10/group)				Dose of black ginseng extract (mg/kg) (female; n = 10/group)			
	0	500	1000	2000	0	500	1000	2000
*Hematology*								
WBC (10^3^/mm^3^)	10.7 ± 3.5	10.7 ± 4.2	11.3 ± 2.0	11.1 ± 1.4	8.6 ± 2.6	10.1 ± 3.5	7.4 ± 2.5	10.3 ± 2.0
RBC (10^6^/mm^3^)	7.7 ± 0.4	7.7 ± 0.2	7.8 ± 0.3	7.8 ± 0.3	7.2 ± 0.3	7.2 ± 0.4	7.2 ± 0.3	7.0 ± 0.4
HGB (g/dl)	15.3 ± 1.5	15.5 ± 0.8	15.5 ± 0.6	15.8 ± 0.5	14.9 ± 0.3	14.8 ± 0.3	14.5 ± 0.6	14.5 ± 0.8
HCT (%)	44.0 ± 1.7	43.9 ± 1.7	43.9 ± 1.3	44.8 ± 1.6	40.8 ± 1.1	41.1 ± 1.5	40.6 ± 1.3	40.3 ± 1.7
PLT (10^3^/mm^3^)	961.0 ± 100.9	940.0 ± 105.5	954.0 ± 142.4	931.0 ± 50.2	910.0 ± 83.7	890.0 ± 96.8	984.0 ± 81.6	1054.0 ± 164.5
MCV (fl)	57.1 ± 2.0	57.1 ± 1.6	56.4 ± 1.5	57.5 ± 1.3	57.1 ± 2.4	57.4 ± 3.2	56.2 ± 1.8	57.7 ± 2.4
MCH (pg)	19.8 ± 1.9	20.2 ± 0.8	19.9 ± 0.7	20.3 ± 0.6	20.8 ± 1.0	20.7 ± 1.0	20.1 ± 0.5	20.8 ± 0.9
MCHC (g/dl)	34.8 ± 2.9	35.3 ± 0.9	35.3 ± 0.4	35.3 ± 0.5	36.5 ± 0.6	36.1 ± 0.7	35.7 ± 1.0	36.0 ± 0.5
Neutrophils (%)	10.8 ± 3.3	13.3 ± 4.7	11.5 ± 4.4	14.2 ± 5.4	11.7 ± 4.1	13.9 ± 6.0	12.4 ± 3.8	10.9 ± 4.1
Eosinophils (%)	0.7 ± 0.2	1.1 ± 0.5	0.8 ± 0.2	0.8 ± 0.3	1.1 ± 0.4	1.0 ± 0.2	1.0 ± 0.5	0.9 ± 0.3
Basophils (%)	0.3 ± 0.1	0.3 ± 0.1	0.2 ± 0.1	0.2 ± 0.1	0.2 ± 0.1	0.2 ± 0.1	0.2 ± 0.1	0.2 ± 0.0
Lymphocytes (%)	86.0 ± 3.5	82.6 ± 5.4	85.0 ± 4.3	81.5 ± 5.5	84.9 ± 4.7	82.6 ± 6.0	83.7 ± 4.9	86.0 ± 4.2
Monocytes (%)	1.5 ± 0.5	2.0 ± 0.9	1.6 ± 0.4	2.3 ± 0.4*	1.6 ± 0.6	1.6 ± 0.5	1.9 ± 0.9	1.2 ± 0.3
Reticulocytes (%)	3.0 ± 0.5	3.2 ± 0.4	3.0 ± 0.4	3.3 ± 0.5	3.0 ± 0.5	3.0 ± 1.0	2.9 ± 0.2	4.8 ± 3.6
PT (sec)	9.6 ± 0.5	9.7 ± 0.4	9.8 ± 0.4	9.5 ± 0.7	8.6 ± 0.2	8.6 ± 0.3	8.5 ± 0.3	8.3 ± 0.3
aPTT (sec)	38.3 ± 5.1	32.1 ± 2.0*	33.6 ± 2.0*	32.7 ± 1.5*	29.7 ± 2.1	30.5 ± 2.0	32.2 ± 1.6	31.3 ± 2.3
*Serum biochemistry*								
BUN (mg/dL)	12.9 ± 1.3	12.6 ± 2.1	13.0 ± 1.8	13.3 ± 1.9	14.2 ± 2.0	13.6 ± 1.9	13.6 ± 2.5	14.2 ± 2.3
TC (mg/dL)	71.0 ± 9.1	71.0 ± 8.5	82.0 ± 16.1	72.0 ± 8.5	83.0 ± 14.7	83.0 ± 14.2	84.0 ± 10.0	81.0 ± 11.7
TP (g/dL)	5.6 ± 0.2	5.5 ± 0.3	5.6 ± 0.2	5.4 ± 0.3	5.8 ± 0.3	6.0 ± 0.2	6.0 ± 0.2	6.0 ± 0.3
Albumin (g/dL)	2.3 ± 0.1	2.2 ± 0.1	2.2 ± 0.1	2.2 ± 0.1	2.6 ± 0.2	2.7 ± 0.2	2.7 ± 0.2	2.7 ± 0.3
TB (mg/dL)	0.02 ± 0.02	0.03 ± 0.01	0.03 ± 0.01	0.04 ± 0.01	0.05 ± 0.02	0.05 ± 0.02	0.05 ± 0.01	0.06 ± 0.02
ALP (IU/L)	668.0 ± 98.7	768.0 ± 179.1	694.0 ± 213.1	792.0 ± 199.4	345.0 ± 110.5	348.0 ± 70.0	369.0 ± 107.3	334.0 ± 100.2
AST (IU/L)	105.0 ± 19.8	101.0 ± 18.7	97.0 ± 16.2	88.0 ± 27.0	90.0 ± 16.9	104.0 ± 43.9	91.0 ± 19.6	99.0 ± 34.3
ALT (IU/L)	38.0 ± 5.0	43.0 ± 6.6	40.0 ± 7.6	41.0 ± 2.3	34.0 ± 4.4	42.0 ± 13.9	34.0 ± 4.2	32.0 ± 7.5
GGT (IU/L)	0.5 ± 0.5	0.6 ± 0.5	0.6 ± 0.5	0.5 ± 0.5	0.3 ± 0.5	0.8 ± 0.4*	0.8 ± 0.4*	0.9 ± 0.3*
Creatinine (mg/dL)	0.42 ± 0.04	0.40 ± 0.05	0.37 ± 0.03*	0.37 ± 0.03*	0.41 ± 0.04	0.41 ± 0.04	0.40 ± 0.06	0.38 ± 0.06
TG (mg/dL)	63.0 ± 34.0	67.0 ± 37.0	67.0 ± 39.0	46.0 ± 20.6	38.0 ± 17.0	34.0 ± 16.9	24.0 ± 10.8	29.0 ± 11.4
Glucose (mg/L)	154.0 ± 20.1	149.0 ± 20.9	149.0 ± 22.2	155.0 ± 26.9	161.0 ± 18.4	152.0 ± 15.8	157.0 ± 13.5	142.0 ± 19.9
A/G ratio	0.68 ± 0.06	0.67 ± 0.05	0.67 ± 0.05	0.66 ± 0.05	0.80 ± 0.07	0.79 ± 0.07	0.83 ± 0.07	0.81 ± 0.07
Potassium (mEq/L)	4.93 ± 0.20	4.90 ± 0.24	4.84 ± 0.18	4.79 ± 0.14	4.24 ± 0.23	4.28 ± 0.22	4.15 ± 0.24	4.13 ± 0.30
Chlorine (mEq/L)	103.8 ± 1.0	102.7 ± 3.7	103.5 ± 0.7	102.6 ± 2.5	105.3 ± 1.0	105.2 ± 2.2	105.6 ± 1.9	104.7 ± 1.4
Sodium (mEq/L)	144.5 ± 0.7	142.4 ± 4.3	144.2 ± 0.6	142.5 ± 2.8	143.1 ± 1.2	143.5 ± 2.2	143.4 ± 1.4	143.1 ± 1.3
Calcium (mg/dL)	10.2 ± 0.2	10.1 ± 0.5	10.2 ± 0.2	10.1 ± 0.4	10.0 ± 0.2	10.2 ± 0.3	10.0 ± 0.2	10.1 ± 0.2
Phosphorus (mg/dL)	8.4 ± 0.5	8.2 ± 0.5	8.3 ± 0.5	8.5 ± 0.3	6.6 ± 0.7	6.9 ± 0.7	6.7 ± 0.7	6.9 ± 0.4

*WBC* White blood cells, *RBC* Red blood cells, *Hb* Hemoglobin, *HCT* Hematocrit, *PLT* Platelet, *MCV* Mean corpuscular volume, *MCH* Mean corpuscular hemoglobin, *MCHC* Mean corpuscular hemoglobin concentration, *PT* Partial thromboplastin time, *aPTT* activated partial thromboplastin time, *BUN* Blood urea nitrogen, *TC* Total cholesterol, *TP* Total protein, *TB* Total bilirubin, *ALP* Alkaline phosphatase, *AST* Aspartate aminotransferase, *ALT* Alanine aminotransferase, *GGT* γ-Glutamyl Transferase, and *TG* Triglycerides. *; *p* < 0.05 by one-way ANOVA followed by *post-hoc* Dunnett’s t-test

Serum biochemical analysis (Table 2) revealed a significant decrease of creatinine in the male 1000 and 2000 mg/kg groups in comparison to the vehicle group, while GGT activity in all the female BGE-treated groups was significantly increased. Despite statistical significance detected in comparison with the respective vehicle control groups, these changes were in the normal range according to our historical data (Supplementary Table 2), denying their association with the toxic effects of BGE.

### Gross and histopathological examination of major organs supported safety of black ginseng extract

Measurement of organ weight (Table 3) revealed that the absolute weight of liver and pituitary gland was significantly increased in the female 2000 mg/kg group compared to the vehicle control group, while remarkable difference in the relative weight was observed in the pituitary gland. The same group was found to have a higher ratio of spleen to body weight. These changes were not considered to be BGE-related based on the findings that the changes were observed in our historical ranges of control animals (Supplementary Table 2) and there was no dose-response relationship.

**Table 3:** Absolute and relative weight of major organs from SD rats orally treated with black ginseng extract for 28 days

		Dose of black ginseng extract (mg/kg)			
		0	500	1000	2000
Male (*n* = 10/group)					
Liver	(g)	13.94 ± 0.85	13.03 ± 3.58	13.48 ± 2.53	14.06 ± 1.65
	(g%)	3.47 ± 0.22	3.21 ± 0.91	3.45 ± 0.57	3.54 ± 0.41
Spleen	(g)	0.94 ± 0.64	0.81 ± 0.11	0.72 ± 0.13	0.82 ± 0.12
	(g%)	0.23 ± 0.16	0.20 ± 0.03	0.19 ± 0.03	0.21 ± 0.03
Kidney (R)	(g)	1.46 ± 0.13	1.51 ± 0.19	1.41 ± 0.16	1.47 ± 0.11
	(g%)	0.36 ± 0.03	0.37 ± 0.04	0.36 ± 0.03	0.37 ± 0.02
Kidney (L)	(g)	1.42 ± 0.10	1.46 ± 0.17	1.43 ± 0.13	1.42 ± 0.12
	(g%)	0.35 ± 0.02	0.36 ± 0.03	0.37 ± 0.02	0.36 ± 0.03
Adrenal gl. (R)	(g)	0.024 ± 0.002	0.028 ± 0.006	0.026 ± 0.005	0.026 ± 0.004
	(g%)	0.006 ± 0.001	0.007 ± 0.001	0.007 ± 0.001	0.007 ± 0.001
Adrenal gl. (L)	(g)	0.026 ± 0.005	0.027 ± 0.005	0.025 ± 0.004	0.028 ± 0.003
	(g%)	0.007 ± 0.001	0.007 ± 0.001	0.006 ± 0.001	0.007 ± 0.001
Testis (R)	(g)	1.58 ± 0.13	1.58 ± 0.11	1.52 ± 0.12	1.56 ± 0.16
	(g%)	0.39 ± 0.04	0.39 ± 0.03	0.39 ± 0.04	0.39 ± 0.05
Testis (L)	(g)	1.59 ± 0.13	1.59 ± 0.12	1.49 ± 0.11	1.55 ± 0.17
	(g%)	0.40 ± 0.04	0.39 ± 0.03	0.38 ± 0.04	0.39 ± 0.05
Thymus	(g)	0.47 ± 0.10	0.66 ± 0.51	0.50 ± 0.12	0.43 ± 0.12
	(g%)	0.12 ± 0.02	0.16 ± 0.11	0.13 ± 0.03	0.11 ± 0.03
Heart	(g)	1.31 ± 0.09	1.22 ± 0.25	1.27 ± 0.05	1.28 ± 0.05
	(g%)	0.33 ± 0.02	0.30 ± 0.07	0.33 ± 0.02	0.32 ± 0.02
Lung	(g)	1.39 ± 0.11	1.43 ± 0.11	1.38 ± 0.12	1.47 ± 0.15
	(g%)	0.35 ± 0.02	0.35 ± 0.04	0.35 ± 0.03	0.37 ± 0.03
Brain	(g)	2.04 ± 0.05	1.96 ± 0.19	2.04 ± 0.10	2.06 ± 0.09
	(g%)	0.51 ± 0.02	0.48 ± 0.06	0.53 ± 0.05	0.52 ± 0.03
Pituitary gl.	(g)	0.015 ± 0.005	0.016 ± 0.008	0.012 ± 0.002	0.013 ± 0.004
	(g%)	0.0037 ±0.0013	0.0038 ±0.0019	0.0032 ± 0.0005	0.0032 ±0.0009
Female (*n* = 10/group)					
Liver	(g)	7.95 ± 1.06	8.22 ± 0.87	7.90 ± 0.77	8.38 ± 0.71*
	(g%)	3.35 ± 0.23	3.37 ± 0.26	3.28 ± 0.18	3.44 ± 0.13
Spleen	(g)	0.51 ± 0.05	0.59 ± 0.14	0.50 ± 0.07	0.69 ± 0.26
	(g%)	0.22 ± 0.03	0.24 ± 0.04	0.21 ± 0.03	0.28 ± 0.09*
Kidney (R)	(g)	0.84 ± 0.08	0.85 ± 0.19	0.88 ± 0.11	0.88 ± 0.08
	(g%)	0.36 ± 0.03	0.35 ± 0.07	0.36 ± 0.02	0.36 ± 0.02
Kidney (L)	(g)	0.82 ± 0.07	0.88 ± 0.10	0.86 ± 0.10	0.86 ± 0.08
	(g%)	0.35 ± 0.03	0.36 ± 0.03	0.36 ± 0.02	0.36 ± 0.02
Adrenal gl. (R)	(g)	0.033 ± 0.004	0.033 ± 0.003	0.033 ± 0.005	0.036 ± 0.004
	(g%)	0.014 ± 0.002	0.014 ± 0.001	0.014 ± 0.003	0.015 ± 0.002
Adrenal gl. (L)	(g)	0.035 ± 0.005	0.034 ± 0.004	0.033 ± 0.005	0.036 ± 0.004
	(g%)	0.015 ± 0.002	0.014 ± 0.001	0.014 ± 0.002	0.015 ± 0.002
Ovary (R)	(g)	0.043 ± 0.007	0.043 ± 0.008	0.043 ± 0.010	0.047 ± 0.005
	(g%)	0.018 ± 0.003	0.018 ± 0.003	0.018 ± 0.004	0.019 ± 0.003
Ovary (L)	(g)	0.044 ± 0.010	0.043 ± 0.006	0.041 ± 0.006	0.044 ± 0.004
	(g%)	0.018 ± 0.004	0.018 ± 0.003	0.017 ± 0.003	0.018 ± 0.002
Thymus	(g)	0.41 ± 0.07	0.46 ± 0.07	0.49 ± 0.14	0.52 ± 0.12
	(g%)	0.17 ± 0.02	0.19 ± 0.03	0.20 ± 0.04	0.21 ± 0.04
Heart	(g)	0.87 ± 0.09	0.88 ± 0.07	0.85 ± 0.06	0.87 ± 0.10
	(g%)	0.37 ± 0.02	0.36 ± 0.03	0.35 ± 0.02	0.36 ± 0.02
Lung	(g)	1.05 ± 0.07	1.10 ± 0.08	1.04 ± 0.09	1.10 ± 0.08
	(g%)	0.45 ± 0.04	0.45 ± 0.03	0.44 ± 0.04	0.45 ± 0.03
Brain	(g)	1.90 ± 0.09	1.87 ± 0.09	1.85 ± 0.08	1.92 ± 0.09
	(g%)	0.81 ± 0.06	0.77 ± 0.05	0.77 ± 0.06	0.79 ± 0.04
Pituitary gl.	(g)	0.015 ± 0.001	0.017 ± 0.002	0.016 ± 0.005	0.020 ± 0.004*
	(g%)	0.0062 ± 0.0007	0.0068 ± 0.0009	0.0068 ± 0.0021	0.0082 ± 0.0021*

**Fig. 3:**
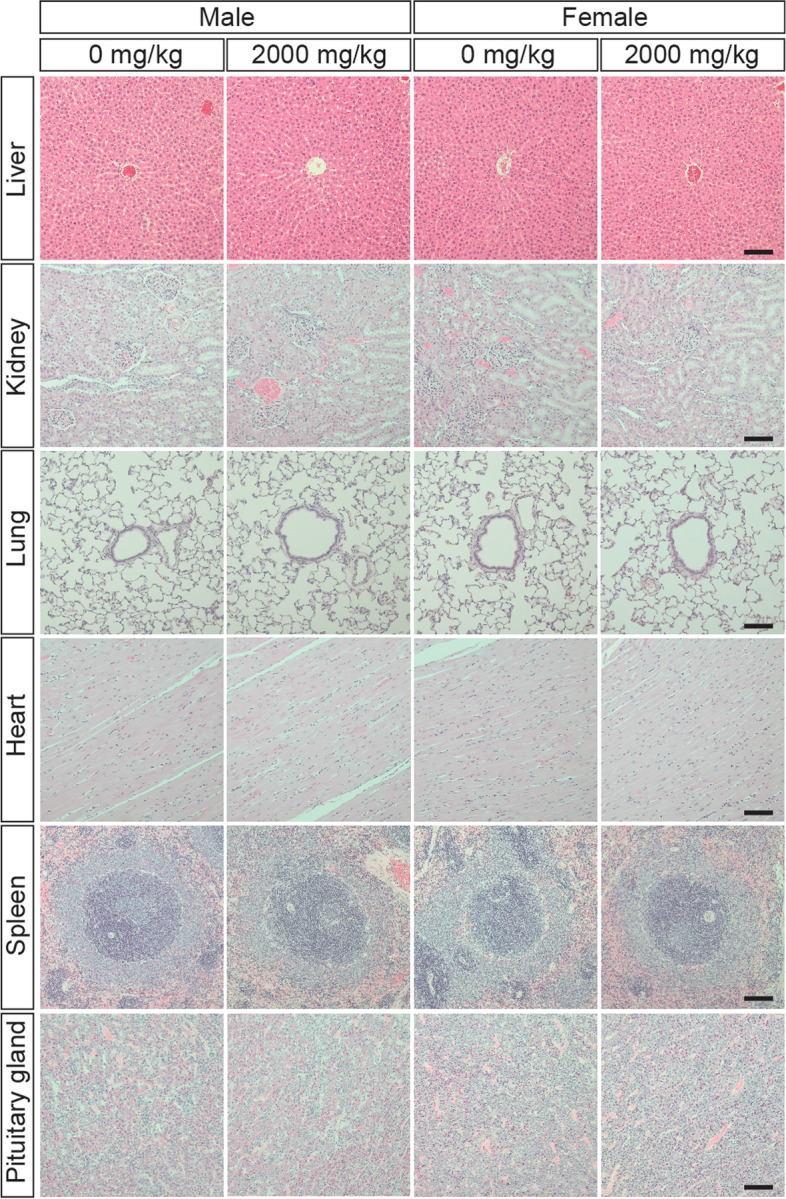
Histopathological examination of Sprague-Dawley rats orally treated with black ginseng extract (BGE) for 28 days. Organs excised at necropsy were sectioned into tissue slices and stained with hematoxylin and eosin after fixation, processing and paraffinization. Representative photomicrographs show that histopathological analysis did not find the test substance-related toxic effects in the organs of male and female 2000 mg/kg BGE treated groups compared to the respective control groups. All images were taken at 10x magnification. Scale bars; 100 μm

Gross examination of major organs at necropsy identified minor changes including discoloration and colored spots in the lung and thymus at a similar rate between the vehicle control and BGE-treated groups (Supplementary Table 3). Besides these, no other macroscopic lesions were observed. Further examination of all major organs using histopathological techniques did not find any pathologically meaningful lesions in relation to administration of the test substance (Fig. 3; Supplementary Table 4).

## Discussion

Ginseng roots have been used as a valuable medicinal herb in oriental medicine from the ancient times and several processing methods have been applied for improvement of medicinal benefits as well as preservation. Since initially developed in Korea, black ginseng has been spread to other countries with recognition of its high pharmacological activities in various diseases. In this study, we investigated subacute toxicity of black ginseng extract in SD rats by oral administration for 28 days.

Prior to the in vivo toxicity study, BGE was assessed for the contents of ginsenosides as well as in vitro cytotoxic activity to confirm the pharmacological validity as a test substance. Consistently with the previous reports [8, 9, 11], HPLC analysis showed efficient accumulation of Rg3 and Rg5 in BGE through the production process. When tested for cytotoxic activity, BGE displayed higher cytotoxicity than RGE in all cell lines tested in the study; BGE showed similarly high potency across the cell lines as indicated by low IC50, whilst the cytotoxic effect of RGE was observed only at the high doses to a lesser degree than BGE.

Anti-cancer activity of black ginseng has been reported in several in vitro and in vivo studies and our results are closely in line with their findings. Black ginseng was shown to elicit cytotoxic activity against several cancer cell lines including MCF-1 human breast cancer, HT-1080 human fibrosarcoma [31], and HepG2 human hepatocellular carcinoma cells [32]. In xenograft mouse models, black ginseng significantly decreased the weight and volume of solid cancer masses derived from Lewis lung carcinoma [31], H22 [33], HepG2 [30, 32], indicating potent anti-cancer activity. Further to these findings, our data showed that BGE is effective in more types of human cancers in vitro, suggesting its potential application in treating a broad range of cancers. It is of great interest to know whether BGE also has such levels of efficacy in vivo, and further studies are warranted to investigate anti-cancer activity of BGE in rodent xenograft models.

Similarity in cytotoxicity patterns observed for Rg5 and BGE in consideration of Rg5 levels suggested that Rg5 is the main contributor to the anti-cancer activity of BGE [16], providing a plausible mechanism underlying the different anti-cancer activity between BGE and RGE. Given the degree of cytotoxicity caused by BGE, Rg3 may play an additive role to the action of Rg5. Several studies reported greater cytotoxicity for Rg5 than Rg3 in a variety of cancer cells including breast cancer cells (e.g., MDA-MB-453 and MCF-7), colorectal cancer cells (e.g., Caco-2 and HCT-8), lung cancer cells (e.g., NCI-H460), hepatocellular carcinoma cells (SMMC-7721) and gastric cancer cells (SGC-7901) [16, 34, 35]. In agreement with these findings, we observed that Rg5 consistently showed greater reduction of cell viability than 20(S)-Rg3 across all the six cell lines at the low doses, confirming its superior cytotoxic potency in a broad-spectrum of cancers. Given the structural similarity to Rg3 except for a hydroxyl group on Carbon-20, how Rg5 can possess remarkably higher therapeutic activity is an intriguing question. Rg3 and Rg5 have been shown to suppress the growth of cancers by influencing diverse aspects of tumor cell biology including cell cycle and proliferation, angiogenesis, metastasis and stemness of cancer stem cells through regulating signaling pathways such as PI3K/PKB [18, 36], STAT3 [35], MAPK [37], C/EBPβ/NF-κB [15], Wnt [38] and SNAIL [39]. Nonetheless, it is still not clear which one(s) among these pathways are responsible for the greater effect of Rg5 or whether other pathways yet to be found exist. Therefore, further investigation into comparison of the degree to which Rg3 and Rg5 activate the known pathways with continuous effort to discover novel pathways may be required to elucidate the molecular mechanism underlying the action of Rg5. Of note, a study reported that upon extracellular treatment, Rg5 accumulated intracellularly in a breast cancer cell line more readily than Rg3, suggesting a possible role of the structural difference in transmembrane transportation of ginsenosides [40].

Although toxicity information on black ginseng, as opposed to the accumulated reports on its pharmacological efficacy, has been limited, a growing body of evidence suggested its safety for oral consumption. A recent study on acute oral toxicity of black ginseng extract reported normal clinical signs, body weight gain, hematology and serum biochemistry without a test substance-associated mortality and histopathological changes, establishing the oral LD50 to be > 15 g/kg [27], which is categorized as practically non-toxic according to Hodges and Sterner Scale [41]. Rg3, one of the enriched ginsenosides in BGE, has been reported to be relatively safe for oral consumption through several toxicity studies; LD50 of 20(S)-Rg3 was determined to be > 800 mg/kg in SD rats and > 1600 mg/kg in mice, and NOAEL in SD rats was 180 mg/kg [42]. In another study using beagle dogs, NOAEL of 20(S)-Rg3 was detected to be 20 mg/kg BW after oral administration for 26 weeks [23]. Regarding Rg5, 30-day repeated intraperitoneal administration of 20 mg/kg into mice did not cause any significant deviations from normal ranges in all clinical and pathological parameters examined in the study [18]. The same research group also found that mice injected with 40 mg/kg of Rg5 showed marked size reduction of engrafted gastric tumors with normal findings in body weight gain during 30 days as well as histopathological analysis [37]. In fact, the levels of Rg3 and Rg5 in our BGE fell in the dose ranges tested in these studies, and our study consistently revealed that oral administration of up to 2000 mg/kg of BGE for 28 days was not toxic in SD rats, demonstrating the safety of BGE for repeated oral consumption. Furthermore, our findings also indicate the reliability of the BGE manufacturing process as well as other BGE components including minor ginsenosides accumulated through the processing such as compound K. Nonetheless, the toxicity profile of BGE is still incomplete and further investigation on the *in vivo* effect of sub/chronic exposure and genotoxicity studies may be required to understand the whole spectrum of its toxicity with characterization of target organs.

In this study, we checked the pharmacological validity of BGE in vitro and investigated subacute oral toxicity of BGE in SD rats. Our results demonstrated that BGE prepared for this study had potent and broad-spectrum cytotoxic activity with Rg5 as the main effector, and did not cause any recognizable test substance-induced in vivo toxicity in the 28-day repeated oral toxicity test with up to 2000 mg/kg BW in SD rats, establishing that NOAEL is > 2000 mg/kg. These findings, together with the previous report on its acute toxicity, demonstrated the safe dose range of BGE (CJ EnerG) in rodents, providing the essential information on safe consumption in human.

## Conclusions

Black ginseng extract (BGE; CJ EnerG), under the conditions employed in this study, demonstrated potent cytotoxic effects to a greater extent than red ginseng extract (RGE) in all the human cancer cell lines tested with its efficacy closely resembling that of ginsenoside Rg5. Evaluation of oral safety in the 28-day repeated-dose toxicity study showed that BGE did not cause any noticeable toxicity related to the test substance in SD rats, establishing its NOAEL to be > 2000 mg/kg BW.

## Supplementary Information

**Additional file 1: Supplementary Table 1.** Urinalysis of SD rats orally treated with black ginseng extract for 28 days. The results of analysis on urine from the SD rats orally treated with black ginseng extract for 28 days. **Supplementary Table 2.** Institutional historical data on the normal range of several parameters in SD rats. Institutional historical control data for several hematological and serum biochemical parameters and organ weight in SD rats. **Supplementary Table 3.** Gross findings in major organs from SD rats orally treated with black ginseng extract for 28 days. Macroscopic findings observed in the necropsy of SD rats orally treated with black ginseng extract for 28 days. **Supplementary Table 4.** Histopathological findings in major organs from SD rats orally treated with black ginseng extract for 28 days. Microscopic lesions observed in the hematoxylin and eosin-stained tissue slides of SD rats orally treated with black ginseng extract for 28 days.

## Data Availability

The datasets used and/or analyzed during the current study have been archived in Department of Experimental Animal Research, Biomedical Research Institute, Seoul National University Hospital, and are available from the corresponding author on reasonable request.

## Abbreviations

A/G: Albumin-globulin
AAALAC: Association for Assessment and Accreditation of Laboratory Animal Care
ALP: Alkaline phosphatase
ALT: Alanine aminotransferase
ANOVA: Analysis of variance
aPTT: Activated partial thromboplastin time
AST: Aspartate aminotransferase
ATCC: American Type Culture Collection
BGE: Black ginseng extract
BUN: Blood urea nitrogen
DMSO: Dimethyl sulfoxide
EDTA: Ethylenediaminetetraacetic acid
FBS: Fetal bovine serum
GGT: γ-glutamyl transferase
HGB: Hemoglobin
HCT: Hematocrit
HPLC: High-performance liquid chromatography
IC50: Inhibitory concentration 50%
LD50: Lethal dose 50%
MCH: Mean corpuscular hemoglobin
MCHC: Mean corpuscular hemoglobin concentration
MCV: Mean corpuscular volume
MTT: Methylthiazolyldiphenyl-tetrazolium bromide
NOAEL: No-observed-adverse-effect-level
OD: Optical density
OECD: Organization for Economic Co-operation and Development
PLT: Platelet
PT: Partial thromboplastin time
PTX: Paclitaxel
RBC: Red blood cell
RGE: Red ginseng extract
SD: Sprague-Dawley
SPF: Specific pathogen free
TB: Total bilirubin
TC: Total cholesterol
TG: Triglyceride
TP: Total protein
WBC: White blood cell
