# Supplementary material for: Efficacy and safety evaluation of black ginseng (Panax ginseng C.A. Mey.) extract (CJ EnerG): broad spectrum cytotoxic activity in human cancer cell lines and 28-day repeated oral toxicity study in Sprague-Dawley rats
Source: BMC Complement Med Ther. 2022 Feb 16;22:44. doi: 10.1186/s12906-022-03522-3 (PMC8848956; doi:10.1186/s12906-022-03522-3)
Supplement: Supplementary file 1 — Additional file 1: Supplementary Table 1. Urinalysis of SD rats orally treated with black ginseng extract for 28 days. The results of analysis on urine from the SD rats orally treated with black ginseng extract for 28 days. Supplementary Table 2. Institutional historical data on the normal range of several parameters in SD rats. Institutional historical control data for several hematological and serum biochemical parameters and organ weight in SD rats. Supplementary Table 3. Gross findings in major organs from SD rats orally treated with black ginseng extract for 28 days. Macroscopic findings observed in the necropsy of SD rats orally treated with black ginseng extract for 28 days. Supplementary Table 4. Histopathological findings in major organs from SD rats orally treated with black ginseng extract for 28 days. Microscopic lesions observed in the hematoxylin and eosin-stained tissue slides of SD rats orally treated with black ginseng extract for 28 days. [file 12906_2022_3522_MOESM1_ESM.pdf]

Supplementary Table 1. Urinalysis of SD rats orally treated with black ginseng extract for 28 days

Parameter	Value	Dose of black ginseng extract (mg/kg)							
		Male (n=5/group)				Female (n=5/group)			
		0	500	1000	2000	0	500	1000	2000
Specific gravity		1.025 ±0.015	1.029 ±0.011	1.030 ±0.007	1.033 ±0.013	1.023 ±0.018	1.026 ±0.013	1.019 ±0.006	1.025 ±0.017
Glucose (mg/dl)	Negative	5/5	5/5	5/5	5/5	5/5	5/5	5/5	5/5
Ketone	Negative	1/5	1/5	0/5	0/5	3/5	3/5	5/5	3/5
	Trace	2/5	1/5	2/5	2/5	2/5	2/5	0/5	1/5
	15	2/5	3/5	3/5	1/5	0/5	0/5	0/5	1/5
	40	0/5	0/5	0/5	2/5	0/5	0/5	0/5	0/5
pH	6.0	0/5	0/5	0/5	0/5	1/5	0/5	0/5	0/5
	7.0	0/5	0/5	0/5	1/5	0/5	0/5	1/5	0/5
	7.5	0/5	0/5	0/5	0/5	0/5	1/5	0/5	0/5
	8.0	0/5	0/5	1/5	1/5	2/5	0/5	1/5	1/5
	8.5	4/5	5/5	4/5	3/5	2/5	3/5	2/5	3/5
	9.0	1/5	0/5	0/5	0/5	0/5	1/5	1/5	1'/5
Leukocytes	Negative	4/5	3/5	4/5	1/5	5/5	5/5	4/5	4/5
	Trace	1/5	2/5	1/5	4/5	0/5	0/5	1/5	1/5
Nitrite	Negative	5/5	5/5	4/5	5/5	5/5	5/5	5/5	5/5
	Positive	0/5	0/5	1/5	0/5	0/5	0/5	0/5	0/5
Protein (mg/dl)	Negative	0/5	0/5	0/5	0/5	3/5	3/5	3/5	3/5
	Trace	4/5	1/5	1/5	2/5	1/5	0/5	2/5	1/5
	30	0/5	4/5	4/5	2/5	1/5	1/5	0/5	1/5
	100	1/5	0/5	0/5	1/5	0/5	1/5	0/5	0/5
Urobilinogen (E.U./dl)	0.2	4/5	4/5	4/5	2/5	3/5	3/5	5/5	4/5
	1.0	0/5	1/5	1/5	3/5	2/5	2/5	0/5	1/5
	2.0	1/5	0/5	0/5	0/5	0/5	0/5	0/5	0/5
Bilirubin	Negative	5/5	5/5	5/5	5/5	5/5	5/5	5/5	5/5
Blood	Negative	5/5	4/5	4/5	5/5	5/5	5/5	5/5	5/5
	Trace-lysed	0/5	1/5	1/5	0/5	0/5	0/5	0/5	0/5

Supplementary Table 2. Institutional historical data on the normal ranges of several parameters in Sprague-Dawly rats

Item	Unit	Gender	Data (n=55, 2006~2018)		
			Range	Min	Max
% Monocytes	%	Male	2.8 ± 1.5	0.8	6.5
aPTT	Sec	Male	26.7 ± 9.0	14.1	46.3
Creatinine*	mg/dL	Male	0.5 ± 0.09	0.34	0.70
GGT	IU/L	Female	0.3 ± 0.48	0.0	1.0
Liver	g	Female	7.6 ± 1.0	5.0	10.3
Pituitary gland	g	Female	0.015 ± 0.002	0.009	0.019
Spleen	g%	Female	0.24 ± 0.03	0.18	0.33
Pituitary gland	g%	Female	0.0063 ± 0.0009	0.0037	0.0085

*; n=45, 2008~2018

Supplementary Table 3. Gross findings in major organs from SD rats orally treated with black ginseng extract for 28 days

Organ	Findings	Dose of black ginseng extract (mg/kg)							
		Male (n=10/group)				Female (n=10/group)			
		0	500	1000	2000	0	500	1000	2000
Lung	Normal	8/10	9/10	10/10	9/10	9/10	10/10	9/10	9/10
	Discoloration	0/10	0/10	0/10	0/10	1/10	0/10	0/10	1/10
	Spots	2/10	1/10	0/10	1/10	0/10	0/10	1/10	0/10
Thymus	Normal	10/10	10/10	10/10	10/10	10/10	10/10	8/10	9/10
	Discoloration	0/10	0/10	0/10	0/10	0/10	0/10	2/10	1/10
Clitoral gland	Normal	-	-	-	-	10/10	9/10	10/10	9/10
	Spots	-	-	-	-	0/10	1/10	0/10	1/10

Supplementary Table 4. Histopathological findings in major organs from SD rats orally treated with black ginseng extract for 28 days

Organ	Findings	Dose of black ginseng extract (mg/kg) Male (n=10/group) 0	Male 2000	Female (n=10/group) 0	Female 2000
Nervous system					
Brain	Normal	10/10	10/10	10/10	10/10
Spinal cord	Normal	10/10	10/10	10/10	10/10
Sciatic nerve	Normal	10/10	10/10	10/10	10/10
Ocular system					
Eyes	Normal	6/10	4/10	4/10	4/10
	Mild vacuolar optic nerve degeneration	4/10	6/10	5/10	6/10
	Mild bilateral peripheral retina degeneration	-	-	1/10	-
Haderian glands	Normal	10/10	10/10	9/10	10/10
	Cellular infiltration	-	-	1/10	-
Digestive system					
Tongue/larynx	Normal	10/10	10/10	10/10	10/10
Salivary glands	Normal	10/10	9/10	10/10	10/10
	Mild hyperplasia	-	1/10	-	-
Esophagus	Normal	10/10	10/10	10/10	10/10
Stomach	Normal	7/10	4/10	4/10	6/10
	Edema	1/10	-	3/10	1/10
	Mild glandular dilation	3/10	3/10	-	1/10
	Mild cellular infiltration	2/10	4/10	5/10	3/10
Duodenum	Normal	10/10	10/10	10/10	10/10
Jejunum	Normal	10/10	10/10	10/10	10/10
Ileum	Normal	10/10	10/10	10/10	10/10
Cecum	Normal	10/10	10/10	10/10	10/10
Colon	Normal	10/10	10/10	10/10	10/10
Rectum	Normal	7/10	7/10	10/10	9/10
	Edema	3/10	3/10		1/10
Liver	Focal inflammation	10/10	10/10	10/10	10/10
	Focal fatty change	3/10	5/10	7/10	9/10
	Diffuse fatty change	1/10	-	-	-
	Extramedullary hematopoiesis	1/10	-	-	4/10
	Necrosis	2/10	6/10	2/10	1/10
	Apoptosis	2/10	1/10	-	-
Pancreas	Normal	10/10	10/10	10/10	10/10
Immune system					
Thymus	Normal	10/10	10/10	9/9*	10/10
Spleen	Normal	10/10	10/10	10/10	10/10
Cervical lymph ln	Normal	10/10	10/10	10/10	10/10
Mesenteric lymph ln	Normal	10/10	10/10	10/10	10/10

*; Total number is reduced due to the loss during tissue preparation

Supplementary Table 4. Histopathological findings in major organs from SD rats orally treated with black ginseng extract for 28 days (continued)

Organ	Findings	Dose of black ginseng extract (mg/kg)			
		Male (n=10/group)		Female (n=10/group)	
		0	2000	0	2000
Endocrine system					
Pituitary gland	Normal	8/9*	8/10	10/10	9/9*
	Rathke's cleft dilation	1/9*	-	-	-
	Calcification	-	1/10	-	-
	Cyst	-	1/10	-	-
Thyroid glands	Normal	6/10	9/10	8/10	4/10
	Keratinized cyst	4/10	1/10	2/10	6/10
Parathyroid glands	Normal	9/9*	8/8*	8/8*	8/8*
Adrenal glands	Cortical vacuolization	10/10	10/10	10/10	10/10
Respiratory system					
Nasal cavity	Normal	10/10	10/10	10/10	10/10
Trachea	Normal	10/10	10/10	10/10	10/10
Lung	Normal	10/10	9/10	10/10	9/10
	Hemorrhage	-	1/10	-	-
	Inflammation	-	-	-	1/10
Cardiovascular system					
Heart	Normal	8/10	9/10	10/10	9/10
	Cardiomyopathy	2/10	1/10	-	1/10
Urinary system					
Kidneys	Normal	7/10	9/10	5/10	6/10
	Chronic progressive nephropathy	1/10	1/10	2/10	-
	Fibrosis	2/10	-	1/10	-
	Mineralization	-	-	2/10	4/10
Urinary bladder	Normal	-	1/10	-	1/10
	Dilation	10/10	9/10	10/10	9/10
Reproductive system					
Preputial gland /Clitoral glands	Normal	9/10	9/10	9/10	10/10
	Cellular infiltration	1/10	1/10	-	-
	Ductular dilation	-	-	1/10	-
Testes	Normal	10/10	10/10	-	-
Epididymides	Normal	10/10	10/10	-	-
Prostate	Normal	6/10	7/10	-	-
	Atrophy	1/10	1/10	-	-
	Inflammation	3/10	2/10	-	-
Seminal vesicle	Normal	10/10	10/10	-	-
Ovaries	Normal	-	-	10/10	10/10
Uterus	Normal	-	-	8/10	10/10
	uterine endometrium squamous metaplasia	-	-	2/10	-
Vagina	Normal	-	-	10/10	10/10
Musculoskeletal and other systems					
Skin/mammary gl.	Normal	10/10	10/10	9/10	10/10
	Follicular dilation			1/10	
Skeletal muscle	Normal	8/10	10/10	10/10	10/10
	Myofiber degeneration	2/10	-	-	-
Femur /bone marrow	Normal	10/10	10/10	10/10	10/10

*; Total number is reduced due to the loss during tissue preparation
